# Validation of an Endoscopic Fibre-Optic Pressure Sensor for Noninvasive Measurement of Variceal Pressure

**DOI:** 10.1155/2016/1893474

**Published:** 2016-05-23

**Authors:** Bin Sun, De-Run Kong, Su-Wen Li, Dong-Feng Yu, Ging-Jing Wang, Fang-Fang Yu, Qiong Wu, Jian-Ming Xu

**Affiliations:** ^1^Department of Gastroenterology, The First Affiliated Hospital of Anhui Medical University, Hefei, Anhui 230022, China; ^2^Department of Surgery, The First Affiliated Hospital of Anhui Medical University, Hefei, Anhui 230022, China

## Abstract

In this study, the authors have developed endoscopic fibre-optic pressure sensor to detect variceal pressure and presented the validation of* in vivo* and* in vitro* studies, because the HVPG requires catheterization of hepatic veins, which is invasive and inconvenient. Compared with HVPG, it is better to measure directly the variceal pressure without puncturing the varices in a noninvasive way.

## 1. Introduction

Variceal pressure is not only a major predictive factor for variceal bleeding, but also a useful parameter to evaluate the effect of pharmacotherapy of portal hypertension [1]. Therefore, it is important to regularly measure variceal pressure for cirrhotic patients. The conventional method of determining variceal pressure is directly puncturing the varix by using a fine needle [2]. This method needs to be improved because it was invasive and has the high risk of bleeding. Several attempts have been taken to develop a noninvasive or minimally invasive measurement, but none of them has been successful due to their inconvenience and impracticability in use [3–8]. We recently developed an endoscopic fibre-optic pressure sensor (EFPS) to measure variceal pressure [9]. Compared with conventional methods of measuring variceal pressure, EFPS is much easier to place the pressure sensor on the suitable varices through the endoscopic biopsy channel. In this study we tested the accuracy and variability of EFPS* in vitro *using a varix model and* in vivo* using canine mesenteric veins that are comparable in size to esophageal varices.

## 2. Material and Methods

**Figure 1:**
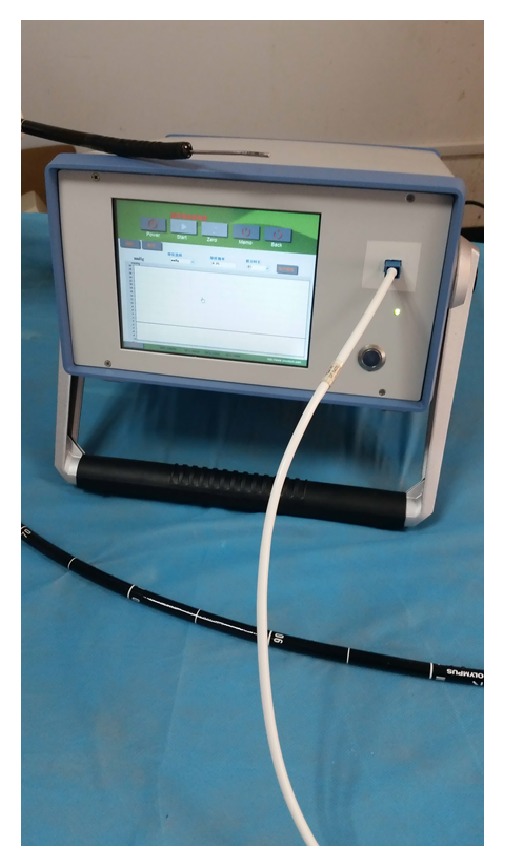
Noninvasive measurement of variceal pressure using the endoscopic fibre-optic pressure sensor inserted through the working channel.

**Figure 2:**
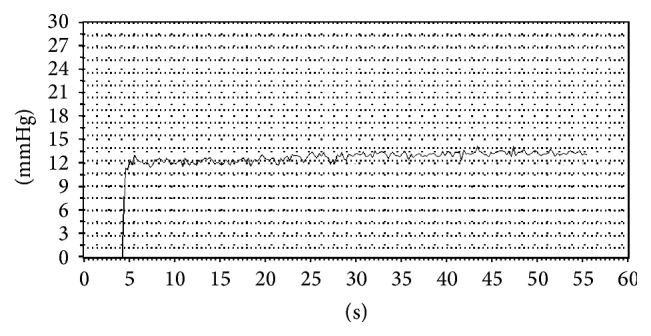
Variceal pressure recording by EFPS* in vitro* study.

*Measurement of Variceal Pressure Using the Endoscopic Fibre-Optic Pressure Sensor (EFPS)*. EFPS was manufactured by Jiuhong Medical Instrument Co., Ltd. (Changzhou, China). Variceal pressure measurement by using EFPS has been described previously [9]. EFPS main unit is connected to a fibre optical that ends in a miniature fibre-optic pressure sensor (FOP-F125, FISO Technologies Inc., Quebec, Canada). When the endoscope is introduced into the distal end of the esophagus, the fibre-optic pressure sensor is compressed by the retrograde insertion through the working channel on the varix with endoscopic control (Figure 1). However, we noticed that the sensor needs to be held by hand so that EFPS connects well with the varix during thewhole process. The pressure tracing detected by EFPS was shown as a sharp upward and stable plateau curve (Figure 2). For the* in vitro* studies, EFPS also showed a plateau phase that is a stable line. However, the major difference between the* in vivo* study and the* in vitro* study by EFPS is that the pressure tracing has periodic fluctuations in accord with breathing variation when used* in vivo*. During the experimental procedure, 2 independent measurers need to be blinded to the pressure in the artificial varix and in the actual mesenteric vein.

### 2.1. *In Vitro* Study

**Figure 3:**
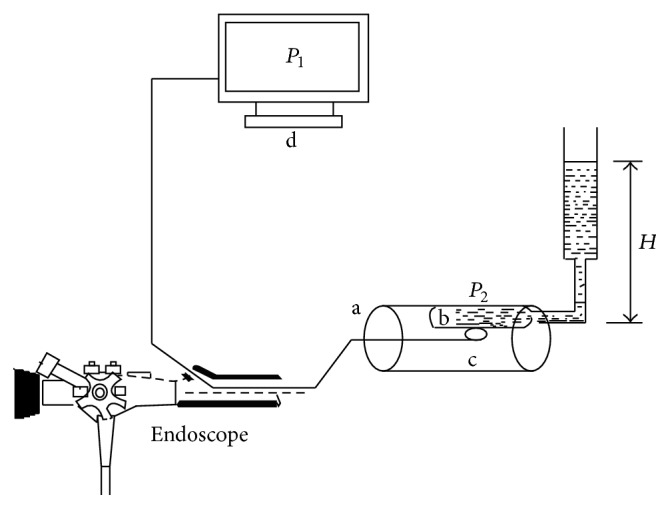
Experimental model of an esophageal varix for comparison of the endoscopic fibre-optic pressure sensor with direct measurement of artificial intravariceal pressure: a: esophageal model; b: artificial varices filled with water and connected to a water column; c: fibre-optic pressure sensor that was inserted through the working channel and compressed on the varix gently; d: polygraph. The pressure in the balloon (*P*_1_) was recorded by polygraph of endoscopic fibre-optic pressure sensor. The pressure in the artificial varix (*P*_2_) was measured using the height of the water column (*H*).

Previously, we have developed an artificial variceal model with a diameter of 4 or 8 mm and a length of 5 cm. It was made from 0.2 mm thick latex and was fixed to the inner wall of an artificial esophagus (diameter: 2.8 cm) [8]. These artificial varices are filled with water and then connected to a liquid column manometer (5 cm in diameter); its height determines the artificial variceal pressure; the zero reference point was established at the level of the middle of artificial varix. The sensor was gently compressed on the artificial varix to measure the artificial variceal pressure (Figure 3).

The height of the liquid column representing the artificial variceal pressure was recorded by one operator, while the other operator that was blinded to the artificial variceal pressure was recording the measuring pressure by the EFPS. In each experiment, 5 measurements were performed in random order for any pressure (increased by 2 cm H_2_O at one time) varied from 6 to 40 cm H_2_O.

### 2.2. Animal Study

#### 2.2.1. Ethics Statements

This study was carried out in strict accordance with the recommendations in the Regulations for the Administration of Affairs Concerning Experimental Animals approved by the State Council of People's Republic of China. The research protocol was approved by the Ethics Committee of Anhui Medical University.

#### 2.2.2. Animals

Ten mongrel dogs, weighing 13.8–16.7 kg (average 15.2 ± 0.9 kg), were used in this experiment. All dogs were purchased from the Laboratory Animal Center of Anhui Medical University. Animals were housed in appropriate stainless steel cages with the size of 0.80 m × 0.80 m × 1.00 m. All cages were placed in an air-conditioned room; the recommended temperature and humidity were 20°C–25°C and 40%–70%, respectively. The cages were washed twice daily. In addition, during the cage washing, the dogs were taken outside for exercise to reduce their stress. The light cycle was controlled using an automatic timer (12-hour light, 12-hour dark). All animals were provided with 600 g of solid food per day with free access to water. Dogs were allowed at least 7 days to adapt to their home environment before experiments. Dogs were considered to be fine based on medical history, physical examination, and laboratory test. The health of the dogs was monitored every week. During this period, all animals stayed healthy, with no clinical or pathological abnormalities based on the report from the veterinary examinations.

Before the operation, these dogs were starved for 12 hours. After anaesthetized with pentobarbital sodium (30 mg/kg) initially, these dogs were weighted. With the dogs' supine, the limbs were fixed in the operation table with the bandages, and the respiration and heart rates were monitored by a multiparameter monitor. All animals were breathing spontaneously during surgery and maintaining the anesthetization. The skin of abdomen was sterilized with 3% iodine and 75% alcohol. Laparotomy was performed by right epigastric incision to expose and isolate the portal vein, superior mesenteric vein, and the inferior vena cava (IVC). A 7-F indwelling catheter that was filled with saline solution and linked to a manometer was maintained in the proximal branch of the superior mesenteric vein, which was at the same level as the portal vein. The mesenteric venous pressure was measured continuously by Philips SureSigns VM8 multiparameter monitor. The zero point was set as the ventral surface of the IVC.

**Figure 4:**
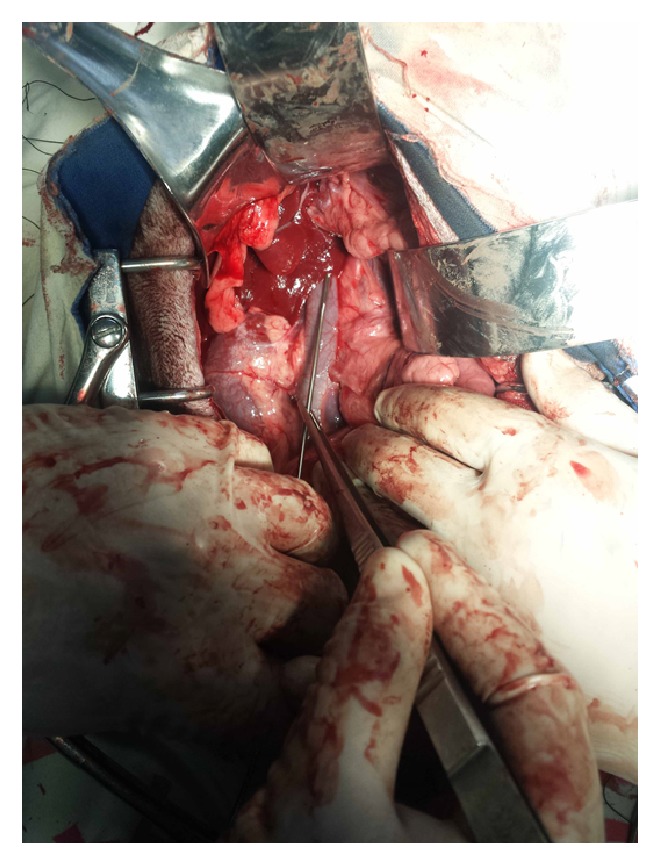
Portal vein pressure of dog was measured by directly compressing the fibre-optic-sensor on the exposed portal vein.

After the portal vein pressure (PVP) was determined, the sensor pressures measured by EFPS were determined in each dog by direct compressing of the sensor on the exposed portal vein (Figure 4). In practice of animal experiments, in order to make a good contact of the sensor with the varix, the fibre-optic pressure sensor was held by hand and directly compressed on the portal vein. All pressure tracings were obtained using EFPS and recorded on a Yoto polygraph (Yoto Technologies Co., Hefei, China).

Two operators performed each experiment. The sensor was gently compressed on the vein by one operator and the other set the zero reference point prior to the application of the sensor on the vein and assessed the quality and stability of the tracing as well as recorded sensor pressure. Each operator was unaware of the placement of the sensor or the sensor pressure recording.

**Figure 5:**
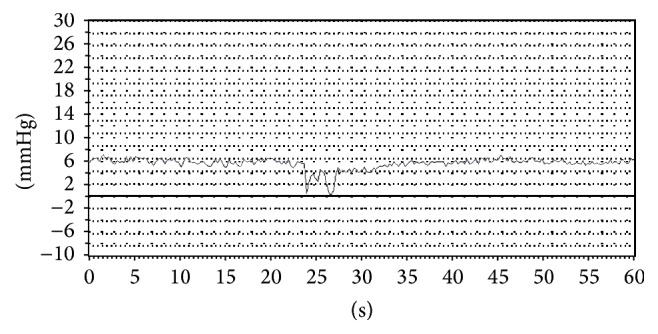
Variceal pressure recording by EFPS in animal study.

The criteria required for a satisfactory measurement included a zero reference tracing and a variceal pressure tracing with stable plateau for at least 10 seconds, with the sensor being maintained on the vein under direct view (Figure 5). The mean value of two satisfactory measurements determined the variceal pressure. At the end of the study the dogs were euthanized with an excess of anesthetic.

### 2.3. Clinical Study

Between December 2013 and April 2014, EFPS was assessed in eight cirrhotic patients (5 men and 3 women; mean age 53.5 years; etiology of cirrhosis for eight patients was hepatitis B virus) who are scheduled for transjugular intrahepatic stent-shunt (TIPS). All had a history of episodes of esophageal variceal bleeding and presented large esophageal varices (F2, F3) under endoscopy. Exclusion criteria included portal vein thrombosis, previous endoscopic treatment of varices (sclerotherapy or endoscopic band ligation), previous surgical portosystemic shunt or an intrahepatic portosystemic stent-shunt, hepatocellular carcinoma or other malignancy, severe clotting defects, and hepatic encephalopathy grade III or IV.

**Figure 6:**
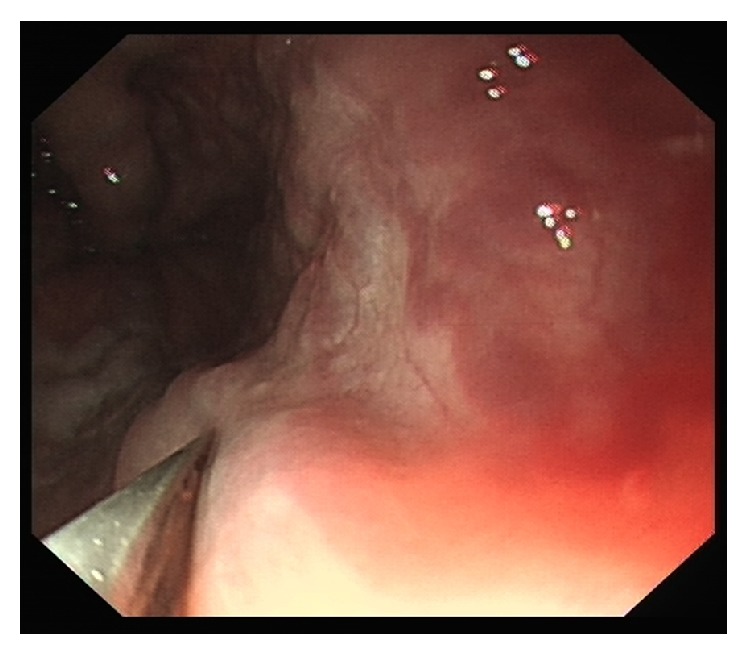
The variceal pressure of patient was measured by EFPS which was gently pressed onto the variceal surface under direct visual control.

**Figure 7:**
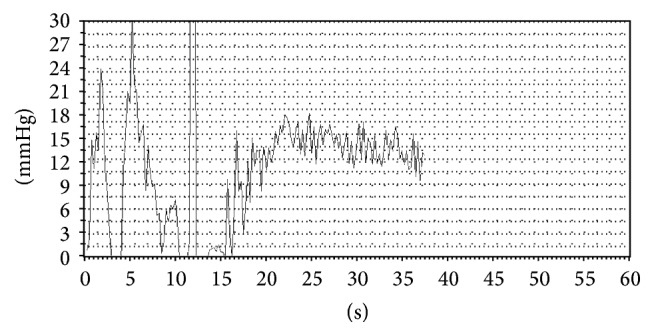
Endoscopic variceal pressure recording showed the satisfactory variceal pressure tracing (note the fluctuations following cardiac and respiratory cycles as well as the stability of the tracing).

Before variceal pressure measurement started, the patients were sedated with intravenous premedication (diazepam 5 mg; butylbromide scopolamine 20 mg), to reduce as much as possible excessive esophageal contractions. First, the endoscope was introduced into the distal esophagus. Then the fibre-optic-probe was protruded to the tip of endoscope through the working channel; the largest varix at the level of the distal portion of the esophageal varices was chosen for measurement (Figure 6). Between peristaltic waves, the probe was gently compressed on the largest varix and lasted for 5 seconds in order to obtain a stable variceal pressure tracing. The criteria required for a satisfactory measurement included a zero reference tracing and a variceal pressure tracing with stable plateau for at least 5 seconds (Figure 7). The mean value of two satisfactory measurements determined the variceal pressure.

Portal pressure was measured by insertion of a 7-F curved catheter into the portal vein connected to an electromanometric transducer during TIPS placement, which was performed within 24 hours after variceal pressure measurement. The difference between portal vein pressure and inferior vena caval pressure was recorded as portal pressure gradient (PPG). The midchest was used as the external zero reference. Pressure tracing with stable plateau lasting for at least 10 seconds was considered satisfactory.

### 2.4. Statistical Methods

Quantitative data were expressed as mean ± standard deviation (SD) and were compared using Student's *t*-test if the data were normally distributed. The correlation between variables was analyzed using the Pearson linear correlation test. Statistical analysis was done using the SPSS 10.0 software package. *P* < 0.05 was considered to be statistically significant.

## 3. Results

### 3.1. *In Vitro* Study

A total of 180 fibre-optic-sensor pressure measurements were obtained successfully by two operators after a brief training.

**Figure 8:**
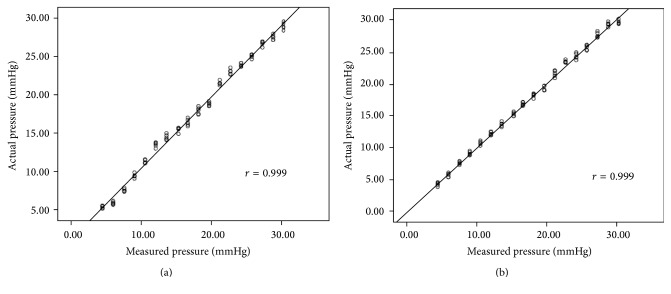
Correlation between measured pressure and the actual intraluminal pressure for model varices of different diameters: (a) 8 mm and (b) 5 mm.

**Figure 9:**
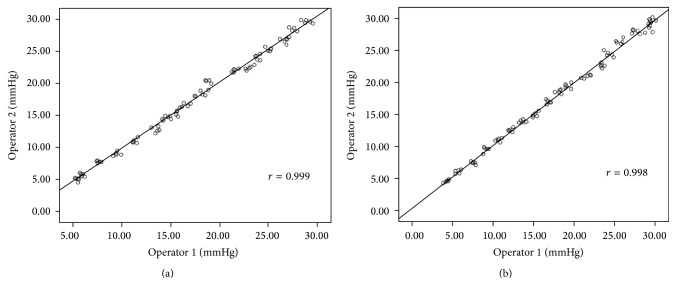
The correlation coefficient between the operators for the artificial intravariceal pressures: (a) 8 mm and (b) 5 mm.

Linear regression analysis showed a good correlation between fibre-optic-sensor pressure and actual intraluminal pressure in the variceal model for different diameters (*r* ≥ 0.998, *P* < 0.001; Figure 8). For the varix models with the diameter of 5 cm, the correlation coefficient between the fibre-optic-sensor pressure and actual intraluminal pressure for Dr. Sun was *r* = 0.999. The correlation coefficient between the fibre-optic-sensor pressure and actual intraluminal pressure for Dr. Li was *r* = 0.999. For the varix models with the diameter of 8 cm, the correlation coefficient between the fibre-optic-sensor pressure and actual intraluminal pressure for Dr. Sun was *r* = 0.999. The correlation coefficient between the fibre-optic-sensor pressure and actual intraluminal pressure for Dr. Li was *r* = 0.998. The correlation coefficient between the operators (Dr. Sun and Dr. Li) for the artificial intravariceal pressures was *r* = 0.998 (Figure 9).

### 3.2. Animal Study

**Figure 10:**
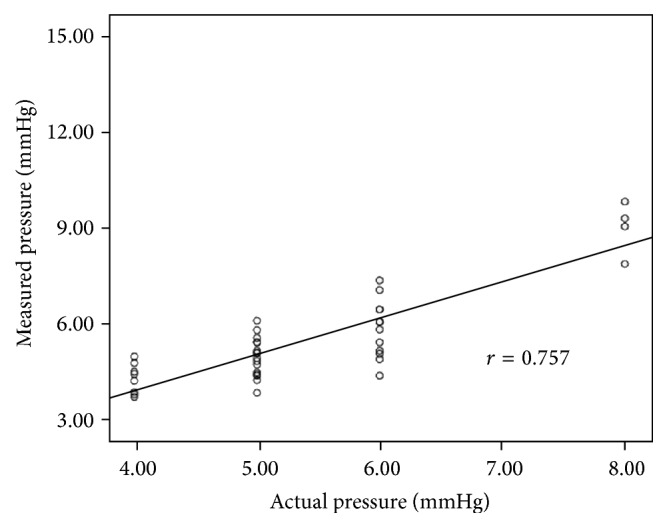
Correlation between measured pressure by EFPS and the actual portal vein pressure in animal study.

Determination of fibre-optic-sensor pressure was technically successful in all of the session. The tracing obtained by fibre-optic-sensor conformed to the tracing criteria stated above for acceptance. We noticed that the selection of satisfactory fibre-optic-sensor pressure tracings was done without the result of actual mesenteric venous pressure. There was a linear correlation between fibre-optic-sensor pressure and PVP values (*r* = 0.757, *P* < 0.001, Figure 10).

### 3.3. Clinical Study

Variceal pressure measurements in eight patients were technically successful. There was no difficulty in measuring the variceal pressure because of easy positioning of the fibre-optic-probe on the varix. The pressure tracing detected by EFPS conformed to the criteria stated above. No adverse effect related to the variceal pressure measurement was observed, especially no variceal bleeding during the measuring procedure. After the patients were given intravenous premedication with diazepam and butylbromide scopolamine, the esophageal peristalsis or breathing that would cause the excessive esophageal peristalsis and then affect the variceal pressure measurement was significantly decreased. We also found that the periods between two peristaltic waves were suitable for obtaining the satisfactory pressure tracing, because the esophagus was at rest during the session.

**Figure 11:**
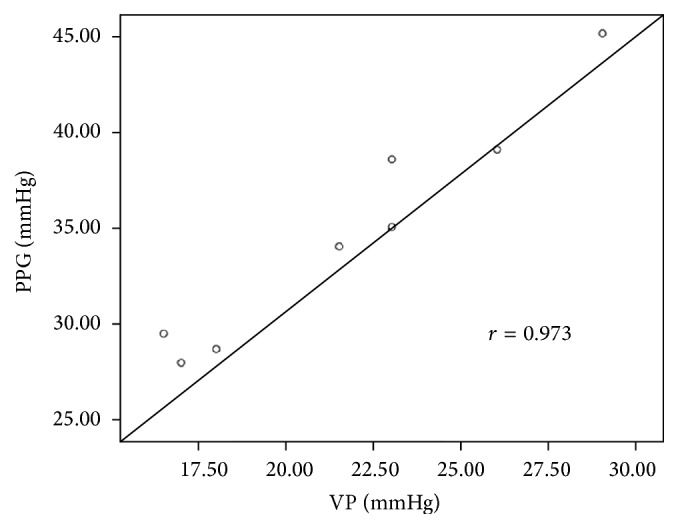
Correlation between the variceal pressure (VP) and the portal pressure gradient (PPG) in cirrhotic patients.

In eight patients, the mean PPG value was 34.7 ± 5.9 mmHg, and mean variceal pressure value was 21.8 ± 4.4 mmHg. There was a linear correlation between variceal pressure and PPG values (*r* = 0.973, *P* < 0.001, Figure 11). It was obvious that PPG values were higher than the variceal pressure.

## 4. Discussion

The variceal pressure can be measured either via directly puncturing the varix by using a fine needle or with noninvasive methods. In recent years, our team described a new noninvasive technique for measuring the variceal pressure. Compared with previous noninvasive technology of measuring variceal pressure, it was much easier to place the fibre-optic-sensor (diameter 2 mm) on the varices through the endoscopic biopsy channel and set up a good contact between the sensor and the vessel [9]. In order to be used clinically, variceal measurements obtained with this device must be accurate and reproducible. Therefore, we testified the reliability and reproducibility of this method* in vitro* and* in vivo*.

In this study, we showed that the variceal pressure measurement using fibre-optic-sensor did not require any specialized training in addition to operating EFPS and standard endoscopy. Like the operation of the esophageal mucosal biopsy, the fibre-optic-sensor is easy to operate for measuring the variceal pressure with an endoscopic control by an operator as described in our previous study [9]. The pressure tracing obtained with EFPS was characterized by a sharp upstroke and stable plateau phase during both* in vitro* and* in vivo* studies. By* in vitro* study, linear regression analysis demonstrated a good correlation between the fibre-optic-sensor and the direct intraluminal pressure measurements for different diameters of artificial varices. More importantly, the coefficient of variance showed that the results of fibre-optic-sensor manometry were also reliable and reproducible.

In the animal study, we also observed a positive correlation between the mesenteric venous pressure and PVP in experimental dogs. We attributed these results to the fact that the diameter of the measuring surface of the fibre-optic-sensor was less than the diameter of these vessels. This may lead to a good contact between the fibre-optic-sensor and the vessel, thereby enabling pressure measurement to obtain a stable plateau phase. In clinical study, the varices of F2 to F3 in patients may be related to the high rate of success. We found that variceal pressure measured by EFPS correlated significantly with the PPG, which indicated the results of EFPS were reliable. As the previous studies showed, the portal vein pressure was higher than the variceal pressure, because of the prevariceal resistance in collateral vessels [10]. However, the clinical study that enrolled a small group of cirrhotic patients probably indicated the preliminary result. In order to further verify the accuracy of EFPS, larger studies with more cases of cirrhosis are underway.

To our knowledge, this fibre-optic-sensor is the smallest gauge to measure variceal pressure, which can be retrogradely inserted through the working channel of an endoscope. In prior literature, a pressure-sensitive gauge attached to the tip of the endoscope was used to measure the variceal pressure. This noninvasive method was first developed by Rigau et al. and continuously improved by others. However, the reliability of the endoscopic gauge* in vivo* remained to be tested because ideal positioning of the gauge with diameter of 5.5 mm on the varix may be difficult and may result in variable readings, especially for the measurement in small varix [3, 11, 12]. In addition to the diameter of gauge, the measurement of variceal pressure by using the conventional pressure-sensitive gauge was likely to be affected by peristalsis in the esophagus, which would make pressure measurement more difficult [11–13]. With the endoscope insertion and air insufflations, the esophageal contractions would increase more and more frequently even though the patients received intravenous premedication such as diazepam and butylbromide scopolamine to reduce possible excessive esophageal movement [9, 13].

Owing to the thermal stability problems that occurred in fibre-optic-sensor, the measured variceal pressures are susceptible to temperature. Before each measurement, the zero reference of fibre-optic-sensor must be set in order to acclimatize to the temperature at the time.

In conclusion, our results indicate that EFPS has the potential to measure variceal pressure* in vivo*, which is independent of variceal size. Further studies in patients using this fibre-optic-sensor are now undertaken to determine the clinical applicability of this device. We believe that this new device may help provide a simple and accurate method for determining variceal pressure, an important parameter related to the risk of initial variceal bleeding and the effectiveness of pharmacologic therapy.
